# Common Myna Roosts Are Not Recruitment Centres

**DOI:** 10.1371/journal.pone.0103406

**Published:** 2014-08-14

**Authors:** Manaswini Sarangi, Payel Ganguly, Zenia, Chiti Arvind, Abhilash Lakshman, T. N. C. Vidya

**Affiliations:** Evolutionary and Organismal Biology Unit, Jawaharlal Nehru Centre for Advanced Scientific Research, Bangalore, India; University of Regina, Canada

## Abstract

We studied communal roosting in the Common Myna (*Acridotheres tristis*) in the light of the recruitment centre hypothesis and predation at the roost. The number and sizes of flocks departing from and arriving at focal roosts were recorded over a two year period. We also recorded the sizes and behaviour of foraging flocks. We found that flock sizes of birds departing from roosts at sunrise were larger than those at the feeding site, suggesting that there was no recruitment from the roosts. Flocks entering the roosts during sunset were larger on average than those leaving the following sunrise, suggesting no consolidation of flocks in the morning. Flocks entering the roosts at sunset were also larger on average than those that had left that sunrise, although there was no recruitment at the feeding site. There was no effect of group size on the proportion of time spent feeding. Contrary to expectation, single birds showed lower apparent vigilance than birds that foraged in pairs or groups, possibly due to scrounging tactics being used in the presence of feeding companions. Thus, the recruitment centre hypothesis did not hold in our study population of mynas. Predation at dawn and dusk were also not important to communal roosting: predators near the roosts did not result in larger flocks, and resulted in larger durations of arrival/departure contrary to expectation. Since flock sizes were smallest at the feeding site and larger in the evening than in the morning, but did not coincide with predator activity, information transfer unrelated to food (such as breeding opportunities) may possibly give rise to the evening aggregations.

## Introduction

Communal roosting is widespread amongst birds (see [1]) and is hypothesised to have evolved due to thermoregulatory benefits [2], anti-predatory benefits [3], or increased foraging efficiency of roost members [1], [4], [5]. While there have been many studies on communal roosting in birds, only a small fraction of them have actually examined communal roosting in the light of these specific hypotheses. Communal roosting in birds is an ancestral trait with multiple losses of the trait across species, as well as secondary origins [6]. A phylogenetic analysis had suggested that increased foraging efficiency could have been an important, although not the only, factor that gave rise to communal roosting, and that secondary losses were indicative of costs to communal roosting [6]. However, phylogenetic analyses would not be able to uncover the actual mechanisms that allowed benefits from communal roosting [6], for which ecological studies are required. We describe a study of communal roosting in the Common Myna (*Acridotheres tristis*, family Sturnidae), in which we primarily tested the “recruitment centre hypothesis” and effect of diurnal predation at the roost.

According to the “recruitment centre hypothesis”, birds actively recruit their roostmates to feeding sites in order to obtain benefits while foraging, either by reducing the probability of predation (through vigilance or the dilution effect) or by increasing the amount of time spent feeding [5], [7]–[8]. This hypothesis was developed in response to the “information centre hypothesis”, which stated that communal roosts had evolved as information centres, where unsuccessful foragers could find knowledgeable foragers to follow to a profitable feeding site the next day [1], [4]. The information centre hypothesis, as originally proposed, was claimed to be group selectionist [8], and the “two-strategies hypothesis” suggested that successful foragers might provide food-related information in exchange for preferred, safe positions within the roost rather than freely advertising information related to the roost or food to unsuccessful birds [9]–[10]. The information centre hypothesis has been generalized to include passive information transfer more recently [11]. Although many studies have investigated information transfer at roosts (for e.g., [12]–[17]), information transfer is also expected under both the recruitment centre and the two-strategies hypotheses [5], being modifications of the information centre hypothesis. In fact, recruitment to food sources was demonstrated in the raven, *Corvus corax* [18]–[20], which also shows information transfer at roosts [20].

Reduced predation at or near the roost (through the dilution effect) could be another benefit of communal roosting [3], [21], and mixed species roosts are thought to support this hypothesis [22]. Gatherings (at pre-roosts) before roosting in the evening are also thought to reduce predation, which might be higher in the evening than in the morning [23].

In the absence of marked birds that might be used to examine information transfer, we made predictions about the recruitment centre hypotheses and (diurnal) predation hypothesis that could be tested using data on flock sizes, predator activity, and foraging behaviour. We predicted that if roosts were recruitment centres and predation near the roost did not affect flock size, 1) flock sizes at the feeding site should be at least as large as flock sizes departing from the roost, 2) average flock sizes at the roost in the morning should be at least as large as those in the evening, and 3) foraging in groups should be more advantageous than foraging alone in terms of increased feeding or decreased vigilance. Prediction two above can be made more specific in two ways, which are not mutually exclusive: a) flocks departing from the roost in the morning should be larger than those that arrived at the roost the previous evening due to recruitment and regrouping of birds, and b) flocks departing from roosts in the morning should be at least as large as those returning the same evening as long as there was no recruitment or diurnal variation in predation at the feeding site. In the second case, average flock sizes may be smaller in the evening because feeding flocks may break up into smaller ones and return separately. This would not been seen if there was recruitment at the feeding site or higher predation during the evening, in which case, average flock sizes would increase in the evening. We also predicted that if predation near or at the roost was important for flocking, 1) flock sizes would be larger while departing from or arriving at the roost, depending on whether predator activity was higher in the morning or evening, 2) the duration of departure/arrival (time between the first and last bird) would be smaller when predation risk was higher, in order to increase the dilution effect, and 3) pre-roosts would function to increase flock sizes before birds flew into the roost if predation was higher in the evening than in the morning.

The common or Indian myna is a commensal species distributed widely across the Indian subcontinent [24]. It forages in open, grassy areas, feeding on insects such as grasshoppers, insect larvae, earthworms, fruit, nectar, and animal remains [25]. These resources are expected to be patchily distributed and unpredictable: for example, grasshoppers show clumped distributions [26] and their population dynamics are complex and difficult to predict [27]. Insect larvae, fruit, nectar, and animal remains are also ephemeral, the first being more abundant after rain, and the latter two being more abundant in certain months. Mynas roost communally at night throughout the year except for females that are incubating or brooding [23]. Nesting is not communal. The annual cycle of the Common Myna consists of a pre-breeding season (November–March), breeding season (April–July), and a post-breeding season (August–October) [28]. The post-breeding season had been characterized by a large number of non-independent juveniles during those months [28], and we retained this classification into three seasons. We chose the Common Myna for this study because it is commensal and usually not shy of being observed, feeds on food that is clumped and ephemeral in nature, which is a condition for recruitment from the roost to operate [7], and roosts communally. Unlike the European starling (family Sturnidae), Common Mynas form much smaller roosts, making them easier to study. Understanding myna behaviour is also of interest in the light of its being one of the world’s most invasive species [29].

## Methods

### Roost counts

The study was carried out from June 2011 to July 2013 in north Bangalore, during the pre-breeding, breeding, and post-breeding seasons. Roost counts were carried out at four roosts located inside the Jawaharlal Nehru Centre for Advanced Scientific Research (JNCASR) campus (13.06897°N, 77.61190°E), which is relatively undisturbed and has fairly high tree cover (see Figure S1). All roosts were bamboo clumps and were selected based on good visibility around them in order to be able to count all the birds flying in or out. There were three larger roosts on campus, at which accurate counts could not be made, and one of which also had crows roosting along with Common Mynas. The four focal roosts (Periphery Roost, Canteen Roost, Gazebo Roost, and Main Building Roost) were used only by mynas although Rosy Starlings (*Sturnus roseus*) sometimes alighted on the roosts but flew away (and were not counted). The focal roosts were not in use throughout the year; therefore, counts were usually carried out only at one or two roosts during a particular season. The times of first and last arrival/departure of birds at the roost were noted, as was the presence or absence of predators in the vicinity of the roost. Mynas started leaving the roost between 04∶20 and 06∶50 hours and started arriving at the roost between 17∶25 and 19∶20 hours, depending on daylight, and observer(s) were present at the roost about an hour prior to departure and about half an hour prior to arrival in case there were any unusually early birds. The area around the roost was scanned for the presence of shikra (*Accipiter badius*), a raptor that hunts adult mynas amongst other prey. We did not see more than one shikra at a roost during any observation. No other potential predator was seen near the roosts. The number and size of flocks arriving at a focal roost at sunset and departing from that roost the following sunrise were counted in 2-minute intervals initially, but subsequently in 30 second intervals from December 2011. Counts were done by a single observer usually, from the terrace of a building adjacent to the roost or from the ground in the case when mynas could enter the roost from limited directions due to the positioning of the roost. New observers counted simultaneously with previous observers until counts matched between observers. We also collected data on flock sizes arriving at and departing from pre-roosts at the JNCASR campus boundary during the post-breeding season of 2012 and the breeding season of 2013. Pre-roosts were tall trees, within about 150 m of roosts, where some mynas would aggregate before flying into a roost. These trees were conspicuous and counts could be carried out accurately. In addition to flock sizes, the direction (based on landmarks in four distinct directions) from which birds entered the pre-roost or in which they left the pre-roost was also noted down.

### Foraging behaviour

We carried out observations on Common Myna foraging in areas around JNCASR, primarily at two new residential layouts, Telecom Layout (∼500 m from JNCASR) and Royal Enclave (∼750 m from JNCASR), and the areas around them. These sites were close to the roosts and we had followed mynas heading from these feeding sites to the roosts in JNCASR. These areas had vacant plots and grassy areas where mynas foraged during the daytime. Mynas also nested (in coconut trees) in the Telecom Layout during the breeding season. We repeatedly and regularly traversed the foraging grounds on foot, looked for shikra and myna presence, and carried out observations on myna foraging behaviour from about 08∶30–12∶30 (called the morning session) and from about 14∶30–17∶00 (afternoon session) during the breeding and post-breeding seasons. We noted down the flock sizes of foraging birds and video recorded foraging behaviour using a *Nikon D5500/Nikon Coolpix L110*/*Canon power shot S3 1S* digital camera. Videos were about 3 minutes long on average as birds usually moved away from the patch after that. Videos were viewed slowed down using the video editing software *Avidemux* version 2.5.6 (http://fixounet.free.fr/avidemux), with a resolution of 1/15 seconds. From each foraging sequence, we calculated the duration of pecking on the ground (as feeding time), the duration of looking around or towards the sky (as vigilance time), and the duration spent walking between bouts of pecking (as searching time) for each bird. If any of the birds moved out of the frame, the remaining bird(s) in the video were not scored during that period. Only one average for the entire flock for each activity was used for the data analysis (see below).

Since there was no manipulation of animals and the study was carried out in non-forest areas, no permit was required. Moreover, the Common Myna is not a protected species (classified as Least Concern by IUCN).

### Data analysis

Based on the bird counts at the roost, we calculated average flock sizes for each sunrise or sunset count, flock size distributions, total roost size, cumulative number of birds that arrived at or departed from the roost at different time intervals, and duration of arrival/departure. In analyses to examine the effect of time of day, season, and diurnal predation on flocking patterns at the roost, average flock size of the flocks seen during one counting session (sunrise or sunset, at one roost) was the experimental unit. Average flock sizes across days were initially considered independent of one another because there was high variability in counts across days, suggesting changes in roost composition (there were several roosts that mynas from the feeding site could fly to on any particular day; visiting different roosts across nights has also been reported by Sonerud et al. [17], [30]). However, we also analysed the data in bins of 3–5 days (see below) so that any possible short-term non-independence of average flock sizes across days would be taken care of. Parametric tests (*t* tests, ANOVAs) were used to compare average flock sizes and durations of arrival/departure across sunrise/sunset, different seasons, and different predation conditions. However, since the flock size distributions themselves were not normal and had long tails, non-parametric tests were used when flock sizes rather than their averages, were compared.

Foraging observations were categorized as feeding alone, paired, or in groups. Average (across individual birds within groups) proportions of time spent in feeding, vigilance, and searching by birds in each observation were logit transformed (see [31]) before being used in two-way ANOVAs, with group size and season as fixed factors. Separate ANOVAs were carried out on the proportions of time spent in different activities because these proportions would not be independent of one another. Statistical analyses were carried out using Statistica 8 [32].

## Results

### Flock sizes at the roost and feeding site

**Figure 1:**
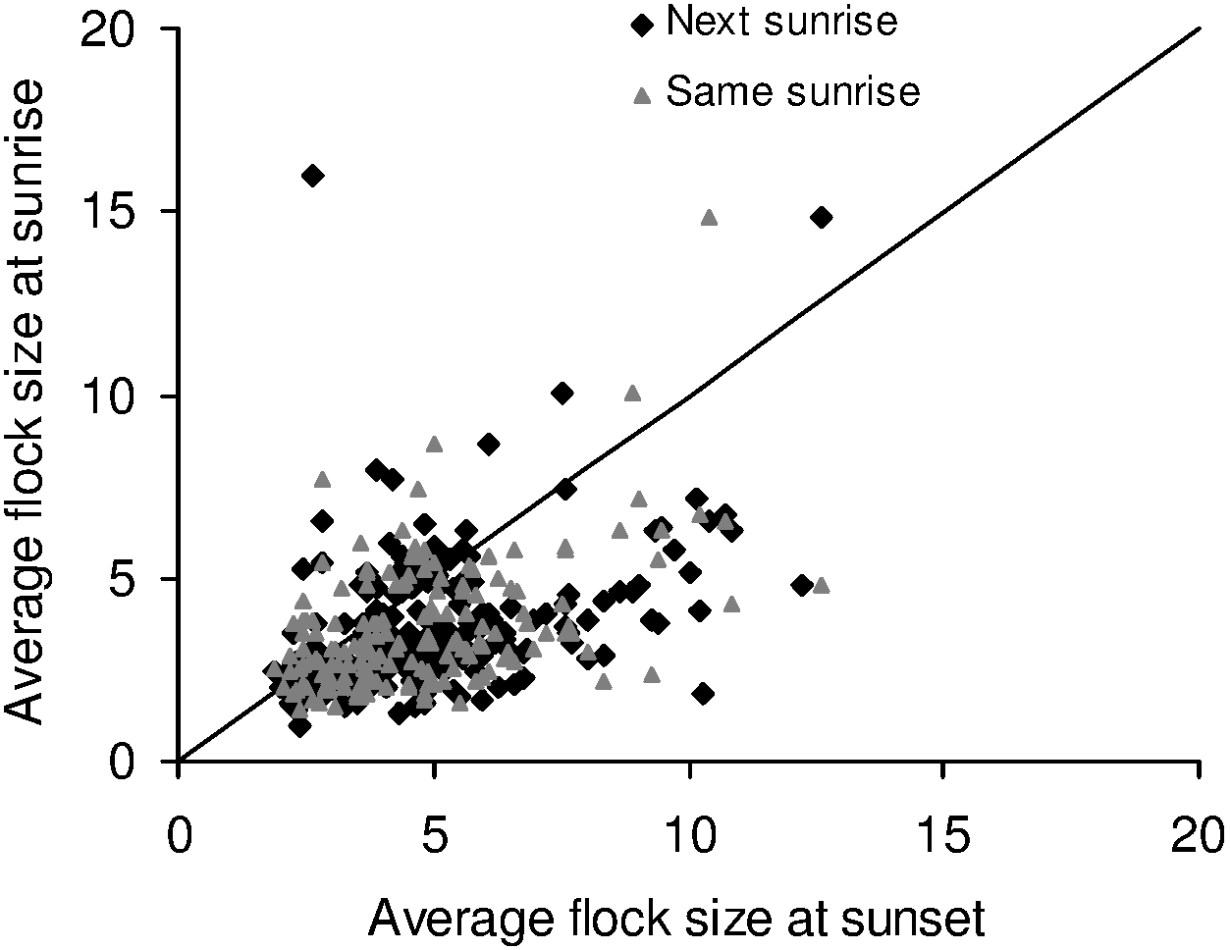
Average flock sizes during sunset and the same or next sunrise across days.

The mean as well as the variance in average flock sizes at sunset (mean, variance = 4.967, 7.245, *N* = 247) were significantly larger than those at sunrise (mean, variance = 3.648, 3.600, *N* = 221) (*t* test with separate variance estimates: *t*_442.49_ = −6.175, *P*<0.001; Levene’s test: *F*_1,466_ = 8.159, *P* = 0.004). This difference in average flock sizes did not accrue from large differences on a small number of days; instead, average flock sizes were larger in the evening than the same morning or the next morning (Figure 1; also see Figure S2, Note S1). The flock size distributions themselves could not be explained by birds flying out randomly from the roost, giving the appearance of different group sizes, because they were significantly different from Poisson distributions (Kolmogorov Smirnov tests: *P*<0.05).

**Figure 2:**
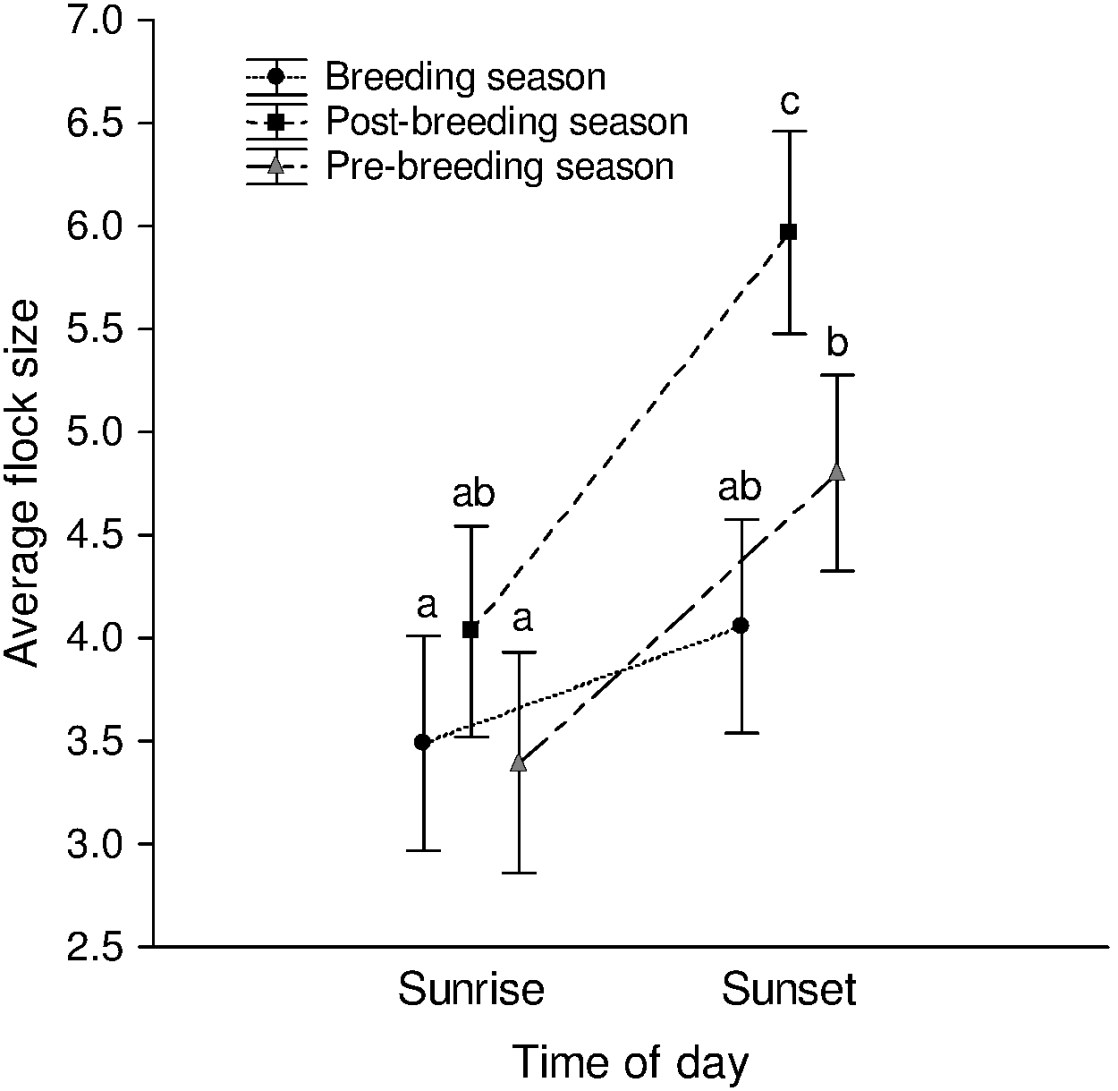
Average flock sizes during sunrise and sunset during different seasons. There was a significant interaction between time of day and breeding season in affecting average flock size (*F*_2,462_ = 3.508, *P* = 0.031) (error bars are 95% CIs about the individual means). Significant pairwise comparisons based on Tukey’s tests are marked with different letters (c>b>a).

An ANOVA using time of day (sunrise/sunset) and season (pre-breeding season/breeding season/post-breeding season) as fixed factors showed that time of day (*F*_1,462_ = 37.884, *P*<0.001), season (*F*_2,462_ = 12.096, *P*<0.001), and the interaction between these factors (*F*_2,462_ = 3.508, *P* = 0.031), to a smaller extent, affected average flock sizes (data from all days of observation, *N*_sunrise_ = 221, *N*_sunset_ = 247). Average flock sizes were significantly larger during the post-breeding season (mean = 5.037) than during the breeding season (mean = 3.775) (Tukey’s HSD test: *P*<0.001) and pre-breeding season (mean = 4.182) (Tukey’s HSD test: *P* = 0.002), but were not different between the pre-breeding and breeding seasons (Tukey’s HSD test: *P* = 0.262). Moreover, average flock sizes were larger during sunset than sunrise during the post-breeding (Tukey’s HSD test: *P*<0.001) and pre-breeding (*P* = 0.002) seasons, but not during the breeding season (Tukey’s HSD test: *P* = 0.654) (Figure 2). Average flock sizes during sunrise did not differ between seasons (*P*>0.05), whereas sunset average flock sizes were larger during the post-breeding season than during the breeding (Tukey’s HSD tests: *P*<0.001) or pre-breeding seasons (Tukey’s HSD test: *P* = 0.010) (Figure 2). The same analyses were carried out on paired observations, either with counts at sunset and the next sunrise at the same roost, or with counts at sunrise and sunset on the same day at the same roost. This was done using a repeated measures ANOVA, with the time of day being the repeated measure and season being a fixed factor. When paired average flock sizes at sunset and the next sunrise were examined, we found the same results as above, with time of day (the repeated measure, *N* = 211 paired days) (*F*_1,208_ = 79.455, *P*<0.001), season (*F*_2,208_ = 13.702, *P*<0.001), and the interaction between these factors (*F*_2,208_ = 9.550, *P*<0.001) affecting average flock size. The same results were also obtained when average flock sizes at sunrise and the same day’s sunset at the same roost were compared (*N* = 166 paired days, time of day: *F*_1,163_ = 59.534, *P*<0.001, season: *F*_2,163_ = 8.809, *P*<0.001, interaction between these factors: *F*_2,163_ = 5.831, *P* = 0.004). In both cases, all the post-hoc tests also gave the same results. We also examined whether the difference in average flock size between sunset and sunrise was dependent on the total roost size, but found that it was not significantly affected by total roost size (Regression of the difference between average flock size at sunset and the next sunrise on total roost size at sunset: *N* = 211 paired days, *R* ^2^ = 0.004, *P* = 0.376; difference between the average flock size at sunset and the same sunrise regressed on total roost size at sunset: *N* = 166 paired days, *R* ^2^ = 0.011, *P* = 0.187).

The difference in average flock sizes between sunrise and sunset did not seem to be due to roost identity. Although only a single roost was occupied or observed during several months of the study, there were two two-month periods when a single observer (the identity of the observer was different in each period) had carried out counts at two roosts, roughly alternatingly. An ANOVA on the average flock sizes using roost identity (Canteen/Gazebo roosts), time of day (sunrise/sunset), and the two periods when different observers had done the counts as fixed factors showed that only the time of day (*F*_1,111_ = 93.356, *P*<0.001) affected the average flock size (effect of roost: *F*_1,111_ = 0.479, *P* = 0.490; effect of time period: *F*_1,111_ = 0.381, *P* = 0.538). There was a third, shorter period of roughly alternating counts at two roosts (of three weeks before one roost started getting abandoned), which was analysed separately because the roost identities were different (Canteen versus Periphery roosts; the Periphery roost started getting abandoned). The ANOVA on data from this comparison again showed only time of day affecting average flock size (*F*_1,33_ = 4.816, *P* = 0.035). However, when about two weeks of low bird count in the Periphery roost (average sunset count of 38.8 birds compared to 85.3 in the preceding weeks) before the roost was abandoned was included, there was additionally an effect of roost (*F*_1,68_ = 6.612, *P* = 0.012) and interaction between roost identity and time of day (*F*_1,68_ = 5.112, *P* = 0.027) on average flock size. This was because the Periphery roost that was being abandoned did not show a difference in average flock size between sunrise and sunset (Tukey’s HSD test: *P* = 0.999) while the Canteen roost did (Tukey’s HSD test: *P* = 0.011).

The difference in average flock sizes across season and time of day were not due to possible non-independence of average flock sizes on consecutive days. We created a dataset with the averages of flock sizes across every three days of counts at the same roost (over a week of actual time in some cases since counts were done on alternate days when two roosts were being monitored at the same time) in order to take care of any short-term non-independence of average flock sizes across days. When this dataset was analysed using a two-way ANOVA, with time of day and season as fixed factors, we found that time of day (*F*_1,151_ = 19.463, *P*<0.001) and season (*F*_2,151_ = 6.277, *P* = 0.002) continued to significantly affect average flock size. Average flock sizes were larger at sunset as before. They were also larger during the post-breeding season than during the breeding season (Tukey’s HSD test: *P* = 0.001) and pre-breeding season (Tukey’s HSD test: *P* = 0.039), but not different between the pre-breeding and breeding seasons (Tukey’s HSD test: *P* = 0.546), as before. Similarly, averages of flock sizes taken in 5-day count bins also showed a significant effect of time of day (*F*_1,88_ = 11.495, *P* = 0.001) and season (*F*_2,88_ = 3.625, *P* = 0.031) on average flock size.

**Figure 3:**
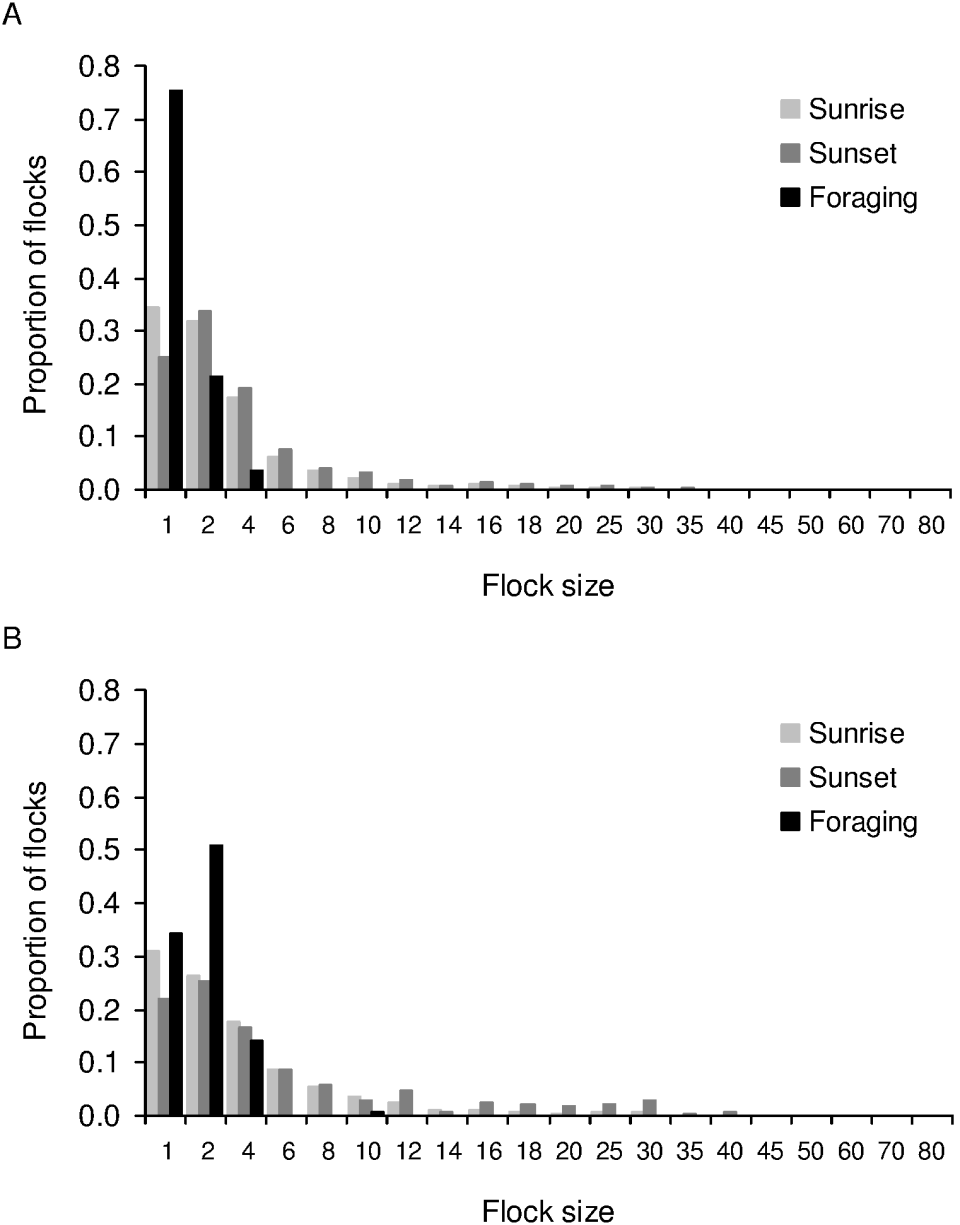
Flock size distributions while entering/leaving the roost and while foraging. Proportions of flocks of different sizes during sunrise (departing from the roost), sunset (arriving at the roost), and while foraging, are shown for the A) breeding season, and B) post-breeding season.

Flock sizes of foraging birds were smaller during the breeding season (mean±1.96 SE = 1.282±0.072, *N* = 202) than the post-breeding season (mean±1.96 SE = 1.892±0.178, *N* = 120) (Kolmogorov-Smirnov test: *P*<0.001). Flock sizes of foraging birds were significantly smaller than flock sizes of birds departing from or arriving at the roost (not average flock sizes at the roost, since there was no equivalent average flock size of foraging birds each day), at sunrise and sunset during the breeding season (Kolmogorov-Smirnov tests: *P*<0.001 for both comparisons, number of flocks: *N_sunrise_* = 9294, *N_sunset_* = 8830, *N_foraging_* = 202), as well as during the post-breeding season (Kolmogorov-Smirnov tests: *P*<0.001 for both comparisons, number of flocks: *N_sunrise_* = 4220, *N_sunset_* = 3003, *N_foraging_* = 120). The proportions of single and paired birds combined were higher during foraging than during sunrise or sunset (Figure 3).

When the effect of the predator on flock size had been included in a complete ANOVA as above (along with time of day and season), there was no effect of the predator on average flock size (*F*_1,456_ = 0.036, *P* = 0.850). We carried out an ANOVA (with predator presence/absence and season as fixed factors) with data from only sunrise roost counts also since shikras were seen infrequently at sunset. This also showed no effect of shikra presence or absence on average flock sizes (ANOVA: *F*_1,215_ = 1.132, *P* = 0.289). The total number of shikra sightings near roosts was 45 during sunrise (out of 223 days of roost observation) and 12 (out of 248 days of roost observation) during sunset. Of these, the number of shikra attacks (shikras flying into the roost, although it is not possible to fly around freely inside the bamboo roost, or flying out at a myna) that we observed was 34 at sunrise and 4 at sunset. We did not see any shikra at the foraging ground. Since the lack of a significant effect of shikra presence could result from the relatively small number of shikra sightings compared to the number of days of observation, we also examined the effect of shikra presence on average flock sizes over shorter periods. Each period was a stretch of observation days during a single season, at a single roost, and at the same time of day (sunrise or sunset), when shikras had been spotted at least four times during the observation days. During one of six such periods (five at sunrise and one at sunset) examined, average flock sizes were larger in the presence (*N* = 10 days) of shikra than in its absence (*N* = 28 days) (*t* test: *t*_36_ = −3.057, *P* = 0.004), but there was no significant difference in average flock sizes between days when shikras were present or absent during the other five periods (*t* tests: *P*>0.05).

### Duration of departure from/arrival at the roost

**Figure 4:**
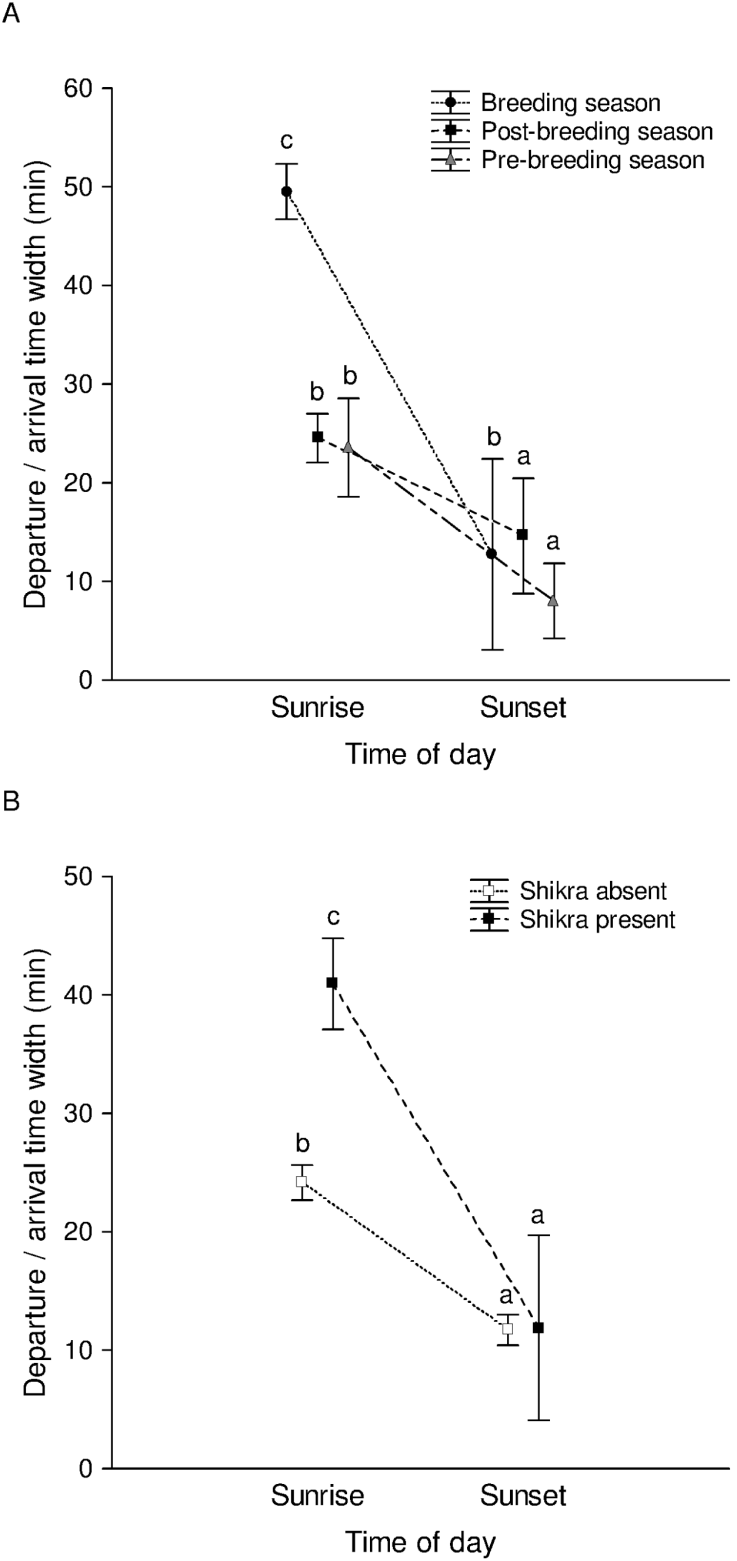
Duration of departure/arrival at sunrise/sunset during different seasons and predator presence/absence. The effect of time of day and season (A) and the effect of time of day and predator presence (B) on the duration of departure from/arrival at the roost (error bars are 95% CIs about the individual means). Significant pairwise comparisons based on Tukey’s tests are marked with different letters (c>b>a).

The times of departure and arrival themselves changed over months depending on the length of day (Figure S3). The effect of various factors on the duration of arrival/departure was examined in a General Linear Model (GLM), with time of day, season, and the presence or absence of shikra as fixed factors, and average flock size and total roost size as continuous predictor variables (data from all days of observation, *N*_sunrise_ = 221, *N*_sunset_ = 247). The duration of arrival/departure depended on the average flock size, total roost size, time of day, season, interaction between time of day and season, shikra presence/absence, and the interaction between time of day and shikra presence/absence (Table 1; multiple *R* ^2^_adjusted_ for the whole model = 0.686). Departure duration at sunrise (mean = 28.4 min) was longer than the arrival duration (mean = 10.8 min) at sunset (post-hoc Tukey’s HSD test: *P*<0.001). The duration of arrival/departure during the breeding season (departure mean = 44.9 min, arrival mean = 17.4 min, overall mean = 31.1 min) was significantly longer than those during the post-breeding (departure mean = 21.0 min, arrival mean = 9.1 min, overall mean = 14.8 min) and pre-breeding (departure mean = 19.1 min, arrival mean = 6.6 min, overall mean = 12.2 min) seasons (Tukey’s HSD tests: *P*<0.001), while durations of arrival/departure during the latter two seasons were not highly significantly different (*P* = 0.048). The difference between the departure durations during the breeding season and the other seasons was especially marked during sunrise (Figure 4, Figure S4). Contrary to expectation, we also found that the duration of arrival/departure was longer in the presence of shikra (mean = 36.4 min) than in its absence (mean = 16.9 min) (Tukey’s HSD test: *P*<0.001). There was also an interaction between shikra presence and the time of day, with the duration of departure at sunrise being longer in the presence of shikra (mean = 43.9 min) than in its absence (mean = 24.7 min) (Tukey’s HSD test: *P*<0.001), but the duration of arrival at sunset not differing in the presence (mean = 11.0 min) or absence (mean = 6.9 min) of shikra (Figure 4). A multiple regression of average flock size and total roost size on the duration of arrival/departure showed a negative effect of average flock size and a positive effect of total roost size on the duration of arrival/departure, although both effects were small (multiple *R* ^2^_adjusted_ = 0.244, P<0.001, β_average flock size_ = −0.340, β_total roost size_ = 0.444) (also see Figure S5).

**Table 1:** Results of the GLM on the duration of arrival/departure of birds at roosts.

Effect	SS	df	MS	F	P
Intercept	16690.22	1	16690.22	174.504	<0.001
Average flock size	4556.60	1	4556.60	47.641	<0.001
Total roost size	8393.15	1	8393.15	87.754	<0.001
Time of day	7837.62	1	7837.62	81.946	<0.001
Season	2468.64	2	1234.32	12.905	<0.001
Predator	1349.04	1	1349.04	14.105	<0.001
Time of day×season	1915.49	2	957.75	10.014	<0.001
Time of day×predator	1277.81	1	1277.81	13.360	<0.001
Season×predator	410.07	2	205.04	2.144	0.118
Time of day×season×predator	448.69	2	224.35	2.346	0.097
Error	43039.59	450	95.64		

### Pre-roost flock sizes

**Figure 5:**
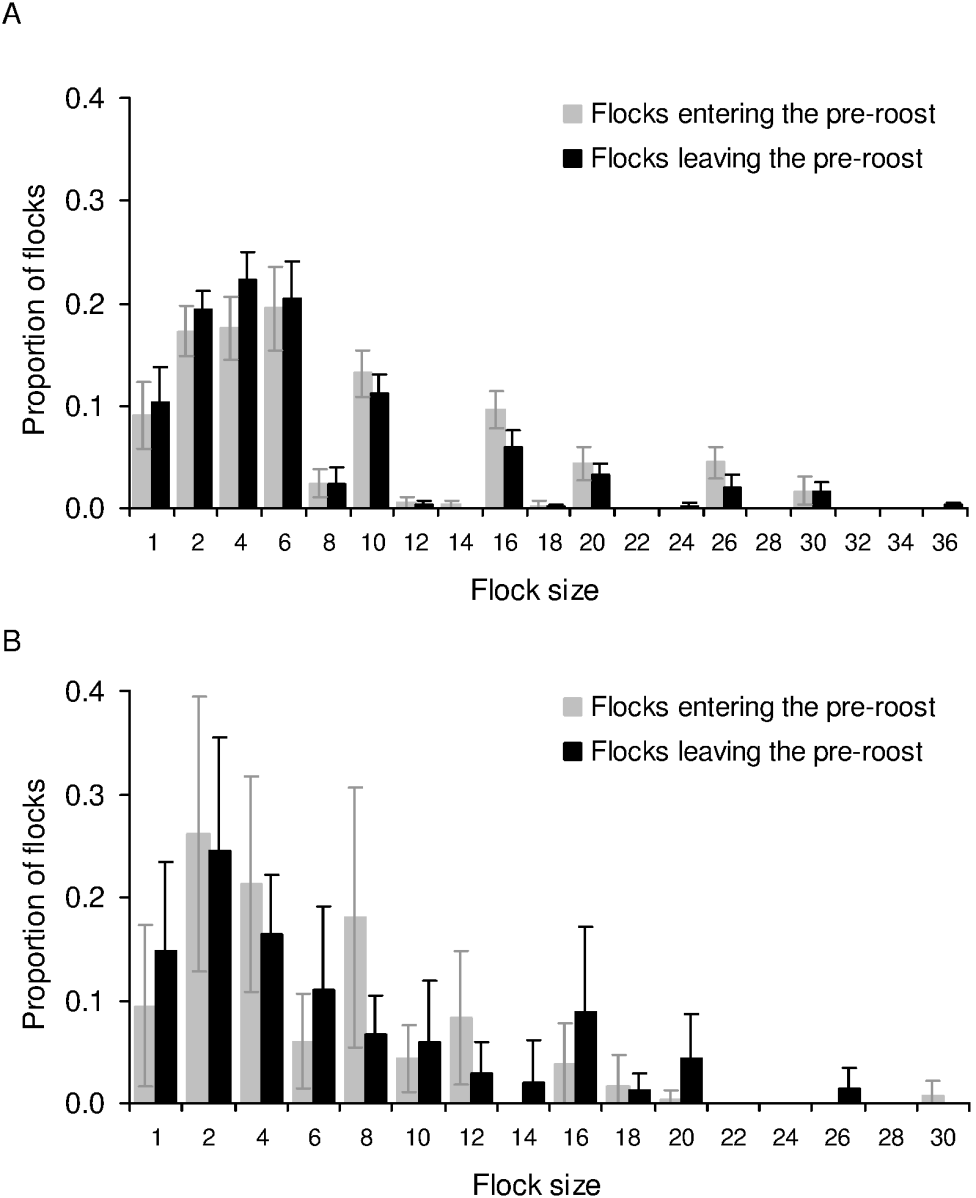
Flock size distributions at pre-roosts. Distributions of sizes of flocks entering and leaving pre-roosts during A) the breeding season, and B) the post-breeding season (error bars are 95% CIs).

We found no difference between the flock size distributions of birds arriving at and departing from the pre-roost (Kolmogorov-Smirnov tests for each day separately: breeding season: 0 out of 11 tests significant, *P* = 1.000; post-breeding season: 1 out of 7 tests significant, *P* = 0.992). Average flock sizes were slightly larger when mynas arrived at the pre-roost (mean±1.96 SE = 7.561±0.783) than when they departed from it (mean±1.96 SE = 6.334±0.574) (paired *t* test: *t*_11_ = 3.571, *P* = 0.005) during the breeding season (Figure 5), but there was no difference during the post-breeding season (mean±1.96 SE = 5.469±0.819 for incoming birds,  = 6.210±1.234 for outgoing birds) (paired *t* test: *t*_7_ = −0.594, *P* = 0.574). These pre-roost flock sizes were similar to the evening flock sizes during the post-breeding season, while they were larger than the evening flock sizes during the breeding season (see Figure 2). Flocks were seen to arrive from different directions to the same pre-roost, and the largest proportion of birds from the same direction varied from 0.74–0.89. Flocks were also seen to depart to different roosts from the same pre-roost and the largest proportion of birds headed towards the same roost varied from 0.40–0.61 during the 2012 post-breeding season to 0.79–0.93 during the 2013 breeding season.

### Foraging behaviour

**Figure 6:**
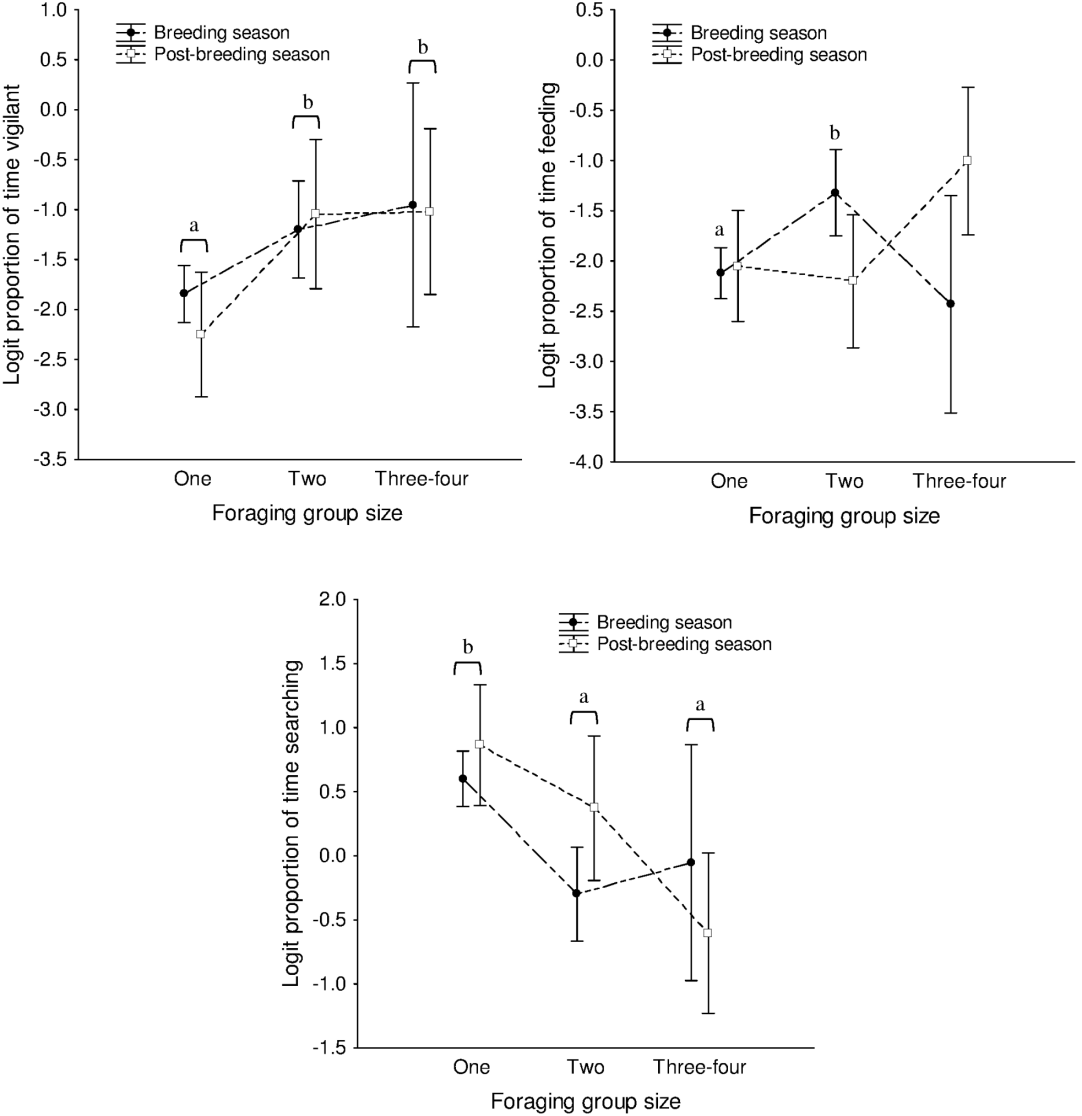
Time spent in different activities while foraging in groups of different sizes. Logit transformed proportions of time spent in feeding, vigilance, and searching by single birds, paired birds, and groups of 3–4 birds during the breeding and post-breeding seasons. Significant pairwise, post-hoc comparisons following a significant effect in the ANOVA are marked with letters (a<b). The horizontal brackets indicate that both breeding and post-breeding seasons have been taken together for the comparison (error bars are 95% CIs about the individual means).

A total of 205 videos from 54 days were analysed to obtain proportions of time spent by each bird (or by each bird on average if two or more birds were foraging together) in different activities while foraging. Two-way ANOVAs carried out on logit transformed proportions (the same effects were significant based on untransformed data also) showed that there was no significant effect of group size on the proportion of time spent feeding (*F*_2,199_ = 1.066, *P* = 0.346), but an effect of group size on the proportion of time spent in vigilance (*F*_2,199_ = 6.865, *P* = 0.001) and searching (*F*_2,199_ = 8.792, *P*<0.001). There was no effect of season on the proportion of time spent in any activity (feeding: *F*_1,199_ = 0.544, *P* = 0.462; vigilance: *F*_1,199_ = 0.114, *P* = 0.736; searching: *F*_1,199_ = 0.291, *P* = 0.590). An interaction between group size and season was found to affect only the proportion of time spent feeding (*F*_2,199_ = 4.705, *P* = 0.010), with paired birds spending a greater proportion of time feeding (mean = 0.211) than single birds (mean = 0.107) in the breeding season alone (Tukey’s HSD test: *P* = 0.019). Post-hoc tests showed that the proportion of time spent in vigilance was lower in birds that foraged singly (mean = 0.128) than those in pairs (mean = 0.240) or groups (mean = 0.269) (Tukey’s HSD tests: single vs pair: *P* = 0.005, single vs group: *P* = 0.037). Single birds spent a significantly greater proportion of their time searching (mean = 0.656) than birds in pairs (mean = 0.475) or groups (mean = 0.394) (Tukey’s HSD tests: *P*<0.001). Pairs and groups of birds did not differ in the proportions of time spent in feeding, vigilance, or searching (Tukey’s HSD tests: *P*>0.05) (see Figure 6).

## Discussion

### Prediction: If roosts were recruitment centres, flock sizes at the feeding site should be at least as large as those that departed from the roost, if predation near the roost did not affect flock size

We found that foraging flocks were smaller than flocks departing from the roost, which was not expected if roosts were recruitment centres. We had followed mynas to check for a match between the feeding site and roosts. Because of the large number of birds in the roosts and absence of unmarked birds, we do not know if and how many birds from other feeding sites were using the focal roosts. However, given our large dataset, the pattern of feeding and roosting flock sizes may not change significantly if we include other feeding sites that we find in the future in our study area. Since mynas arrived and departed from the roosts in larger flocks than those in which they foraged (Figure 3), predation at the time of entering and leaving the roost would seem to be important. However, we found that the presence of predators did not increase flock sizes. Shikras were found lurking near roosts more often in the morning than in the evening, but there was less synchronization amongst mynas in the morning, leading to smaller flock sizes than in the evening. While light-limited foraging time could reduce the variation in arrival times during sunset, the greater synchronization in the evening was not a result of mynas arriving back from the foraging ground just before dark. In fact, many of the birds returned from the foraging ground an hour or half an hour before entering the roost and spent time in the vicinity of roosts or in pre-roosts.

### Prediction: If roosts were recruitment centres and predation did not affect flock size, average flock sizes at the roost in the morning should be at least as large as those in the evening

While a previous study had found that flock sizes were larger during the post-breeding season than the breeding season [28], there had been no previous comparison of morning and evening flock sizes. We found that average flock sizes were larger when mynas returned to the roost at sunset than when they departed at sunrise, during the pre-breeding and post-breeding seasons (Figure 2). This was true of average flock sizes overall, as well as paired evening-next morning, and paired evening-same morning comparisons. Pre-roost flock sizes were similar to the evening flock sizes during the post-breeding season, while they were larger than the evening flock sizes during the breeding season. Therefore, if departing flock sizes were compared with flock sizes arriving at the pre-roosts in the evening, there would be even greater support against the recruitment centre hypothesis. Foraging flocks were smaller than flocks departing from or arriving at the roost, which did not depend on predator presence or absence. Therefore, mynas were not recruiting roostmates to the feeding site. Providing additional food (fruit, kitchen scrap, and insect larvae) at the feeding site led to some of the food being taken by mynas but no local recruitment at the feeding site itself (Manaswini Sarangi, Vijay Ramesh et al., unpublished data). If predator activity varied diurnally at the feeding site, decreasing over the course of the day, feeding flock sizes might decrease during the day, giving rise to the pattern expected based on the recruitment centre hypothesis (with flocks being larger in the morning than those returning to the roost). We did not quantify predation at the feeding site. However, we found that feeding flock sizes were not significantly different between the morning (mean±1.96 SE = 1.514±0.130) and afternoon (mean±1.96 SE = 1.390±0.147) sampling sessions, even when compared separately during the breeding and post-breeding seasons (Tukey’s HSD test following a two-way ANOVA: *P* = 0.196).

There was no effect of roost identity on average flock size in the cases when we were able to test it, except because of a short period of abandonment in one roost. A fuller comparison of roost identities on flock sizes was not possible because of all the roosts not being occupied at the same time. We also do not know if mynas in the large roosts that were not examined behave differently from our focal roosts. It might be useful to try to count at least a small part of the large roost in the future. It might also be useful to examine roosts in the process of being abandoned to see if they show different patterns of average flock sizes across time in general.

### Prediction: Foraging in groups should be advantageous in terms of increased feeding or decreased vigilance

The recruitment centre hypothesis predicts feeding or anti-predation (through vigilance or dilution effects) benefits to birds foraging in groups. We found that the time spent in searching for food was longer for single birds than for pairs or groups, but there was no difference between paired birds and groups in the proportion of time spent in any activity (Figure 6). Further, apart from paired birds spending more time feeding than single birds during the breeding season alone, there was no difference in the proportion of time spent feeding between groups of different sizes. Therefore recruitment of birds to the feeding site is unlikely to be profitable in terms of improving feeding efficiency. This was in keeping with our observation that flock sizes were not large while feeding. We also found that birds foraged predominantly singly during the breeding season, presumably as adults alternated at the nest, and predominantly in pairs during the post-breeding season (Figure 3). It is possible that single birds were not able to increase their feeding time during the breeding season in the absence of a partner. Newey [33] also found that food intake did not change when invasive Common Mynas in Australia fed singly or in groups, and McGiffin et al. [34] found that rates of food intake and vigilance effort did not change significantly across disturbance zones.

Recruitment to the feeding site could also be beneficial where there is increased predation at the feeding site, but we did not observe any shikra at the feeding site, although there were several attacks by shikras at the roosts. The large flock sizes at the roost probably make them a better place to hunt than the open feeding site since shikras are ambush hunters and do not fly after prey in the air for long distances unlike other falcons (M.B. Krishna, pers. comm.). While vigilance per bird is generally expected to be lower in groups than in single birds (for e.g., see [35]–[36]), we found greater vigilance in pairs and groups than in singly feeding birds (Figure 6). This increase in apparent vigilance with an increase in group size could result from birds looking up not to scan for predators, but to look for flushed insects or to look at what their companions were doing while feeding in the presence of conspecifics [37]. Such scrounging would also explain the reduced proportion of time spent in searching by birds in pairs or groups. Interestingly, European starlings (also from the family Sturnidae) were found to spend more time scanning when they were >1 m or >3 m away from each other than when they were about 0.5 m away in an experimental setup [38], and we usually saw mynas foraging about 1 m or more away from one another, possibly due to dispersed food. More detailed behavioural observations on foraging mynas are required to find out whether scrounging is indeed used as a tactic. Recording the traditionally used head-up posture may not be appropriate as a measure of vigilance in such species.

### Predation at the roost

**Figure 7:**
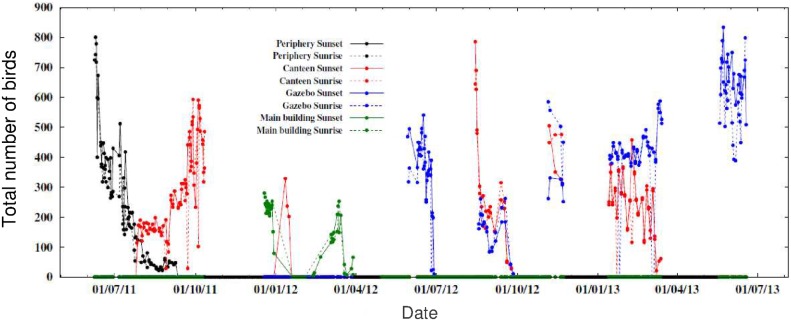
Bird counts across time. Daily variation in the number of birds present at four roosts (Periphery, Canteen, Gazebo, Main building) during sunset and the following sunrise.

The presence of predators did not affect average flock sizes while entering or leaving the roost. It is possible that our selection of roosts that could be clearly monitored led to an upward bias in predator visits because this meant that the roosts were isolated rather than being one continuous large roost. However, despite high predation risk, we found that average flock size was influenced by time of day and season rather than predation risk. We also found that the arrival/departure durations were longer in the presence rather than in the absence of shikra. Mynas seemed to wait inside the roost once they sensed shikra presence and then fly out slowly in their normal flock sizes, rather than flying out together rapidly and creating a dilution effect. It is possible that predators are attracted to communal roosts [39] rather than the mynas roosting communally in order to derive anti-predatory benefits. However, it is also possible that nocturnal predation is important. During regular evening and morning counts at roosts, we found that there were several days when roosts of a few hundred birds counted in the evening would be empty before dawn the next day (Figure 7, Figure S6). Similarly, there were days when there were additional birds at the roost in the morning, which could not be explained by miscounting. Therefore, there was occasional movement between roosts at night. Mahabal and Vaidya [40] had also reported mynas waking up occasionally in the middle of the night, sometimes because of an approaching barn owl or flying fox (not a predator) but usually for unknown reasons. It has also been suggested that mynas and other species that roost along with them might have shared anti-predatory signals at the roost at night [41]. We, therefore, carried out preliminary all-night observations for a week at the Gazebo Roost, from 20∶30–05∶30 hrs, and found two instances (on one night) of a barn owl entering the roost (but flying out immediately), leading to a lot of mynas flying out of the roost at once and some returning back later. More interestingly, we found that the mynas were calling, shuffling, and displacing one another throughout the night, every night. The longest period of uninterrupted silence per night at the roost during the week of all-night observations varied from 8–21 minutes. We plan to carry out a longer study observing these roosts at night to find out whether barn owls are important predators of mynas in the area. It is also not clear at the moment whether anthropogenic lighting in the area (which is not high nevertheless) leads to disturbance, causing birds to keep awake or switch roosts at night.

We also found that mynas aggregated as they returned to the roost as also seen previously by Counsilman [23] and Mahabal [28]. Mahabal [28] found mynas forming pre-roost gatherings only in the non-breeding seasons, while we found pre-roost gatherings during the breeding season also. The few studies of pre-roost aggregations have found that pre-roost aggregations could reduce predation while flying into the roost in large numbers [42] or could be extensions of daytime territory [30] and increase the chance of joining a communal roost [30], [43]. We found that there was no difference in the flock size distributions of birds arriving and departing from pre-roosts. Flocks departing from the pre-roost sometimes further split into smaller flocks while flying to the roost. Therefore, pre-roosts were not serving the function of decreasing predation probability while entering the roost. Moreover, birds from a single pre-roost flew to different roosts, even directly to roosts that were not visible from the pre-roost, suggesting that pre-roosts were not important as vantage points for detecting predators before flying into a roost.

### Information transfer?

It is possible that pre-roost aggregations relate to information transfer since pre-roost flocks were very dynamic and could split and fly to different roosts (see [17], [30], [43]). Individuals would, therefore, be able to sort themselves at these pre-roosts based on their needs for that night or the following day [30]. Since flock sizes were similar at sunrise and sunset during the breeding season, but larger at sunset during the other seasons, there could be information transfer about potential mates during the other seasons when birds aggregate [44]. Birds called while at the pre-roost and also while at the roost at dusk and dawn, and we are currently trying to understand the function of these calls. It is possible that the myna roosts are also information centres without being recruitment centres, due to which flock sizes are larger while leaving the roost than while foraging. Information centres may give rise to flocks of birds since the general directions of food sources may be transmitted [45], perhaps inadvertently (see [11]), even if the conditions at the feeding site do not allow for feeding together in a large flock. Birds will have to be marked in the future to study this.

Flock formation is also thought to arise even in the absence of information transfer due to various reasons [46]. The hypothesis that birds arrive in flocks for some reason and that those that arrive at the roost at the same time depart at the same time because of spending a fixed amount of time at the roost (see [46]) does not hold in our situation because the arrival time width is much smaller than the departure time width. Social bonding into flocks is also unlikely because flocks may split mid-air on the way to foraging grounds. In hooded crows, breeding pairs were found to show more social cohesion, while non-mates showed decreased cohesion and a higher probability of splitting mid-air with increasing distance to the roost [30]. Social facilitation is also thought to give rise to flocks, as is roost size (see [46]). We found that roost size was not a good predictor of average flock size, but we do not know whether social facilitation plays a role in flock formation in mynas.

Unlike flock sizes and arrival/departure width, sizes of our focal roosts were not related to the breeding cycle as there were differences in roost sizes between roosts and within roosts even within the same season (Figure 7) and the pattern of roost size occupancy was not constant across years. There were often unoccupied roosts which had been used previously and were, therefore, potentially suitable as roosts. It is not clear how birds choose one roost over another and playback experiments with myna and predator calls are required to examine this. It had been thought that declining roost sizes would indicate declining resources and that birds would emigrate from the area (see [47]), but in our case, we only saw dramatic size fluctuations and the use of different roosts.

### Other hypotheses

Hypotheses other than the ones we examined include thermoregulation, two-strategies hypothesis, “patch-sitting”, and “conspecific-attraction”. Thermoregulation was not considered a plausible hypothesis because mynas are tropical birds (see [22]–[23]), roosts were found throughout the year and exposed to all cardinal directions, and the temperature of our study area rarely falls below 13°C. According to the patch-sitting hypothesis, roosts are diurnal activity centres that allow birds to be close to rich feeding patches [48]. However, this hypothesis does not explain why birds should roost communally rather than singly unless roosting sites were limited within small areas near feeding sites, and would, therefore, additionally require the recruitment centre hypothesis or two-strategies hypothesis to be invoked. There was no reason to believe that there was a paucity of roosting sites in our study area because there were empty roosts around the year. According to the conspecific-attraction hypothesis, communal roosting allows conspecifics to cluster passively at the feeding sites in the vicinity, facilitating more efficient foraging through local enhancement [49]. This is not likely to be the case in our study as we did not find more efficient foraging in groups. The two-strategies hypothesis may be worth examining once the extent of nocturnal predation is assessed.

Another possible hypothesis is that there are no benefits to roosting communally and that communal roosting is an ancestral trait that persists in the absence of costs. However, roosting communally is generally likely to be costly, in terms of increased conspicuousness to predators [50], increased probability of losing territories in territorial birds that join distant roosts [51], increased transmission or intensity of parasites/pathogens [52]–[53], or deterioration of plumage from droppings [54]. Although we did not measure various costs, and it might be worth doing so in the future, there appeared to be a cost to roosting communally at least in terms of the energy spent competing for space within the roost. The mynas spent half an hour at least, often an hour, squabbling intensely and displacing one another within the roost in the evening. Even after this initial period of settling down in the roost, there were bouts of squabbling and displacement at a lower intensity throughout the night, as revealed from night-long observations. Therefore, it is unlikely that communal roosting in this species is an ancestral trait that happens to persist.

## Conclusion

Overall, our data from flock sizes and foraging do not support the idea of roosts as recruitment centres in the Common Myna. Predation at dawn and dusk also did not explain communal roosting although predation at night remains to be studied. It is plausible that there is information transfer at gatherings before the pre-roosts, at the pre-roosts, or at the roosts. Information transfer could occur in the context of the two-strategies hypothesis if there is significant nocturnal predation or inadvertently [11]. It is also possible that there is information transfer related to breeding opportunities, but further work is required to examine these possibilities.

## Supporting Information

Figure S1 **Locations of roosts and pre-roosts in the study area.** Locations of roosts and pre-roosts in the study area. Roosts and pre-roosts are marked with yellow pins. The Canteen Roost, Gazebo Roost, TSU Roost and Periphery Roost were the focal roosts. The rough areas corresponding to the large roosts are marked as yellow polygons.
(DOC)

Figure S2 **Average flock sizes during sunset and sunrise across days.** Average flock sizes during sunset and the next sunrise (A), and during sunset and the same sunrise (B) across days.
(DOC)

Figure S3 **Time of arrival and departure from roosts.** Time of the first and last bird at the roost during sunrise (primary Y-axis) and sunset (secondary Y-axis) across days (day 0 is 10-June-2011; June has longer daylight hours than other months).
(DOC)

Figure S4 **Percentage cumulative number of birds remaining at the roost at sunrise.** Percentage cumulative number of birds remaining at the roost at sunrise. Each line represent a day of observation.
(DOC)

Figure S5 **Arrival/departure durations, average flock sizes, and roost sizes across days.** Graphs of arrival/departure durations and average flock size or roost size across days to give an idea of variability across days. The time taken by 95% and 100% of the birds to arrive at/depart from the roosts were very similar (100% shown here). These graphs are for the Canteen roost during the post-breeding season of 2011.
(DOC)

Figure S6 **Roost sizes at sunset and the next sunrise.** Scatterplot of the total counts of birds arriving at the roost at sunset and departing the next sunrise. The Spearman rank-order correlation (*R*) between the counts was 0.870 (*P*<0.05, *N* = 211). Note that there were days when there were several hundred birds that entered the roost at sunset but had shifted roost at night and did not emerge the next sunrise. Similarly, there were times when there were additional birds at the roost in the morning.
(DOC)

Note S1 **Average flock size comparisons without outliers.**
(DOC)
