# Supplementary material for: Common Myna Roosts Are Not Recruitment Centres
Source: PLoS One. 2014 Aug 14;9(8):e103406. doi: 10.1371/journal.pone.0103406 (PMC4133212; doi:10.1371/journal.pone.0103406)
Supplement: Figure S1 — Locations of roosts and pre-roosts in the study area. Locations of roosts and pre-roosts in the study area. Roosts and pre-roosts are marked with yellow pins. The Canteen Roost, Gazebo Roost, TSU Roost and Periphery Roost were the focal roosts. The rough areas corresponding to the large roosts are marked as yellow polygons. (DOC) [file pone.0103406.s001.doc]

Figure S1. Locations of roosts and pre-roosts in the study area. Roosts and pre-roosts are marked with yellow pins. The Canteen Roost, Gazebo Roose, TSU Roost and Periphery Roost were the focal roosts. The rough areas corresponding to the large roosts are marked as yellow polygons.
