# Supplementary material for: Common Myna Roosts Are Not Recruitment Centres
Source: PLoS One. 2014 Aug 14;9(8):e103406. doi: 10.1371/journal.pone.0103406 (PMC4133212; doi:10.1371/journal.pone.0103406)
Supplement: Figure S2 — Average flock sizes during sunset and sunrise across days. Average flock sizes during sunset and the next sunrise (A), and during sunset and the same sunrise (B) across days. (DOC) [file pone.0103406.s002.doc]

A

B

Figure S2. Average flock sizes during sunset and the next sunrise (A), and during sunset and the same sunrise (B) across days.
