# Supplementary material for: Common Myna Roosts Are Not Recruitment Centres
Source: PLoS One. 2014 Aug 14;9(8):e103406. doi: 10.1371/journal.pone.0103406 (PMC4133212; doi:10.1371/journal.pone.0103406)
Supplement: Figure S3 — Time of arrival and departure from roosts. Time of the first and last bird at the roost during sunrise (primary Y-axis) and sunset (secondary Y-axis) across days (day 0 is 10-June-2011; June has longer daylight hours than other months). (DOC) [file pone.0103406.s003.doc]

Figure S3. Time of the first and last bird at the roost during sunrise (primary Y-axis) and sunset (secondary Y-axis) across days (day 0 is 10-June-2011; June has longer daylight hours than other months).
