# Supplementary material for: Common Myna Roosts Are Not Recruitment Centres
Source: PLoS One. 2014 Aug 14;9(8):e103406. doi: 10.1371/journal.pone.0103406 (PMC4133212; doi:10.1371/journal.pone.0103406)
Supplement: Figure S4 — Percentage cumulative number of birds remaining at the roost at sunrise. Percentage cumulative number of birds remaining at the roost at sunrise. Each line represent a day of observation. (DOC) [file pone.0103406.s004.doc]

Figure S4. Percentage cumulative number of birds remaining at the roost during sunrise. Each line represent a day of observation.
