# Supplementary material for: Common Myna Roosts Are Not Recruitment Centres
Source: PLoS One. 2014 Aug 14;9(8):e103406. doi: 10.1371/journal.pone.0103406 (PMC4133212; doi:10.1371/journal.pone.0103406)
Supplement: Figure S5 — Arrival/departure durations, average flock sizes, and roost sizes across days. Graphs of arrival/departure durations and average flock size or roost size across days to give an idea of variability across days. The time taken by 95% and 100% of the birds to arrive at/depart from the roosts were very similar (100% shown here). These graphs are for the Canteen roost during the post-breeding season of 2011. (DOC) [file pone.0103406.s005.doc]

|  |
| --- |
|  |
|  |
|  |

Figure S5. Graphs of arrival / departure durations and average flock size or roost size across days to give an idea of variability across days. The time taken by 95% and 100% of the birds to arrive at / depart from the roosts were very similar (100% shown here). These graphs are for the Canteen roost during the post-breeding season of 2011.
