# Supplementary material for: Common Myna Roosts Are Not Recruitment Centres
Source: PLoS One. 2014 Aug 14;9(8):e103406. doi: 10.1371/journal.pone.0103406 (PMC4133212; doi:10.1371/journal.pone.0103406)
Supplement: Figure S6 — Roost sizes at sunset and the next sunrise. Scatterplot of the total counts of birds arriving at the roost at sunset and departing the next sunrise. The Spearman rank-order correlation (R) between the counts was 0.870 (P<0.05, N = 211). Note that there were days when there were several hundred birds that entered the roost at sunset but had shifted roost at night and did not emerge the next sunrise. Similarly, there were times when there were additional birds at the roost in the morning. (DOC) [file pone.0103406.s006.doc]

Figure S6. Scatterplot of the total counts of birds arriving at the roost at sunset and departing the next sunrise. The Spearman rank-order correlation (*R*) between the counts was 0.870 (*P* < 0.05, *N* = 211). Note that there were days when there were several hundred birds that entered the roost at sunset but had shifted roost at night and did not emerge the next sunrise. Similarly, there were times when there were additional birds at the roost in the morning.
