# Supplementary material for: Common Myna Roosts Are Not Recruitment Centres
Source: PLoS One. 2014 Aug 14;9(8):e103406. doi: 10.1371/journal.pone.0103406 (PMC4133212; doi:10.1371/journal.pone.0103406)
Supplement: Note S1 — Average flock size comparisons without outliers. (DOC) [file pone.0103406.s007.doc]

Note S1. Average flock size comparisons without outliers.

As mentioned in the main text, the difference in average flock sizes did not accrue from large differences on a small number of days; instead, average flock sizes were seen to be larger in the evening than the same morning or the next morning (Figure 1, Figure S2). Since maximum flock sizes were also larger during sunset than during sunrise (Paired t tests: sunset vs next day sunrise: t210 = -8.633, P < 0.001; sunset vs same day sunrise: t165 = -6.481, P < 0.001), and the flock size distributions had a long tail, we also compared the average flock sizes during sunrise and sunset after removing all the outliers (points that were 1.5 inter-quartile ranges above or below the upper and lower quartiles, respectively), to see if unusually large flock sizes were contributing to the difference in average flock sizes between sunrise and sunset. Average flock size remained significantly larger and more variable during sunset compared to sunrise even after the removal of outliers (t test with separate variance estimates: t445.47 = -4.349, P < 0.001; Levene’s test: Levene F1,466 = 10.459, P = 0.001).

The ANOVA on average flock sizes using time of day (sunrise / sunset) and season (pre-breeding season / breeding season / post-breeding season) as fixed factors was also repeated on the dataset without outliers. This showed the same results as the ANOVA on all the data, with time of day (*F*_1,462_ = 18.307, *P* < 0.001), season (*F*_2,462_ = 16.102, *P* < 0.001), and the interaction between these factors (*F*_2,462_ = 3.568, *P* = 0.029) affecting average flock sizes.
